# Reliability of a portable device for quantifying tone and stiffness of quadriceps femoris and patellar tendon at different knee flexion angles

**DOI:** 10.1371/journal.pone.0220521

**Published:** 2019-07-31

**Authors:** Guoqian Chen, Jiatao Wu, Guocai Chen, Yanyan Lu, Wei Ren, Wu Xu, Xuemeng Xu, Zugui Wu, Yingxin Guan, Yi Zheng, Bofan Qiu

**Affiliations:** 1 Fifth Clinical Medical School, Guangzhou University of Chinese Medicine, Guangzhou, Guangdong, China; 2 Guangdong Second Traditional Chinese Medicine Hospital, Guangzhou, Guangdong, China; 3 Orthopedics Department, Guangdong Second Traditional Chinese Medicine Hospital, Guangzhou, Guangdong, China; University of Maiduguri College of Medical Sciences, NIGERIA

## Abstract

The reliability of MyotonPRO that can monitor the mechanical properties of tissues is still unclear. This study aimed to analyze the within-day inter-operator and between-day intra-operator reliability of MyotonPRO for assessing tone and stiffness of quadriceps femoris and patellar tendon at different knee angles. The tone and stiffness of healthy participants (15 males and 15 females, aged 24.7±1.6 years) in the supine and resting position were measured using the MyotonPRO device. The measurements were quantified at 0°, 30°, 60°, and 90° of knee flexion. The intraclass correlation coefficient (ICC), standard error of measurement (SEM), and minimal detectable change (MDC) were calculated and a Bland–Altman analysis was conducted to estimate reliability. The results indicated excellent inter-operator reliability (ICC > 0.78) and good to excellent intra-operator reliability (ICC > 0.41). The inter-operator SEM measurements ranged between 0.1–0.9 Hz and 3.8–37.9 N/m, and intra-operator SEM ranged between 0.5–1.3 Hz and 7.9–52.0 N/m. The inter-operator MDC ranged between 0.3–2.5 Hz and 10.5–105.1 N/m, and intra-operator SEM ranged between 1.1–3.3 Hz and 21.9–144.1 N/m. The agreement of inter-operator was better than that of intra-operator. The study concluded that MyotonPRO is a reliable device to detect the tone and stiffness of quadriceps femoris and patellar tendon.

## Introduction

Tone is considered an intrinsic function required to maintain postural stability in balanced equilibrium positions as well as at efficient muscle energy costs [1]. Stiffness is considered an intrinsic ability to resist changes of muscle’s shape caused by an external force [2]. Both the tone and stiffness can be used to assess the health of muscles and tendons [3], and they can be altered under pathological circumstances such as sports-related injuries [4], pain [5], and cramps [6]. Clinicians often use palpatory techniques to assess the tone and stiffness of tissue to guide treatment and appraise treatment effectiveness [7, 8]. However, the reliability of manual palpatory techniques have been criticized because of subjective limitations. Apart from subjective instruments, elastography ultrasound of musculoskeletal ultrasound is widely used to quantify the mechanical properties of tissues[9, 10]. Unfortunately, it is not easy to acquire and operate owing to the equipment costs and required technical expertise. Therefore, it is crucial to quantify the mechanical properties of muscles and tendons in a rapid and reliable way that requires little technical expertise for the convenience of clinical and scientific research.

The MyotonPRO (MyotonPRO, Estonia) is an easy-to-operate, portable, objective, non-invasive, convenient device that can be used to quantify tone and stiffness not only in muscles but also in tendons. The principle is that MyotonPRO elicits oscillations through multiple short impulses from a testing probe, and these oscillation waveforms are reflective of the viscoelastic properties of tissues [11]. Previous studies have demonstrated that the device is reliable in evaluating the viscoelastic properties of various muscles and tendons, including rectus femoris [12, 13], gastrocnemius [2], biceps brachii [13], and Achilles tendons [14] in healthy populations and in people with pathological conditions such as acute stroke [15], chronic spinal cord injury [16], and dementia [17]. However, except the ones with positive results, several authors have raised doubts about the reliability of the device[18].Further, the reliability of MyotonPRO varies in healthy populations of different studies[12, 19].Hence, it is meaningful to perfect reliability research and establish normative data using the MyotonPRO device to provide evidence for future assessments of skeletal muscles and tendons.

The quadriceps femoris and patellar tendon (PT), which are closely related to the functions of the hip and knee joint, are extraordinarily important tissues in the human body. They are essential for daily activities such as walking, running, jumping, and squatting. Quadriceps femoris plays an important role in stabilizing the patella and the knee joint during gait. It is subdivided into four parts: rectus femoris (RF), vastus medialis (VM), vastus lateralis (VL), and vastus intermedius (VI). All four parts ultimately insert into the tuberosity of the tibia via the patella, forming the PT. Apart from VI, the remaining three parts can be considered to be muscular markers on the body surface. Because it is deep inside the RF, VI cannot be measured by MyotonPRO. Previous studies demonstrated that the mechanical properties of quadriceps femoris and PT would be changed by anterior cruciate ligament injury[4, 20], stroke[15], or habitual loading[21]. Further, muscle mechanical properties would be changed when the muscle is at different positions[22, 23]. Therefore, it is necessary to measure its mechanical properties at different positions. To the best of our knowledge, studies [12, 13, 19, 24] have been conducted on the reliability of MyotonPRO in measuring the quadriceps femoris when the knee is extended; however, they have all only focused on measuring the reliability of RF and other parts in the quadriceps femoris(VM and VL) at different knee angles have not been investigated. Furthermore, a previous study[23] found the reliability of RF tone at rest and during active muscle contraction would be changed using computerized muscle tonometer. However, no investigations have been conducted to examine the correlation between the angles of the knee and the reliability of mechanical properties of the quadriceps femoris and PT determined by the MyotonPRO device.

The study aimed to determine 1. the within-day inter- and between-day intra-operator reliability of the tone and stiffness of the quadriceps femoris (RF, VM, VL) and PT measured by MyotonPRO at different angles of the knee joint; 2. the difference in tone and stiffness of certain tissues at different angles of the knee joint.

## Method and materials

### Experimental setup

**Fig 1:**
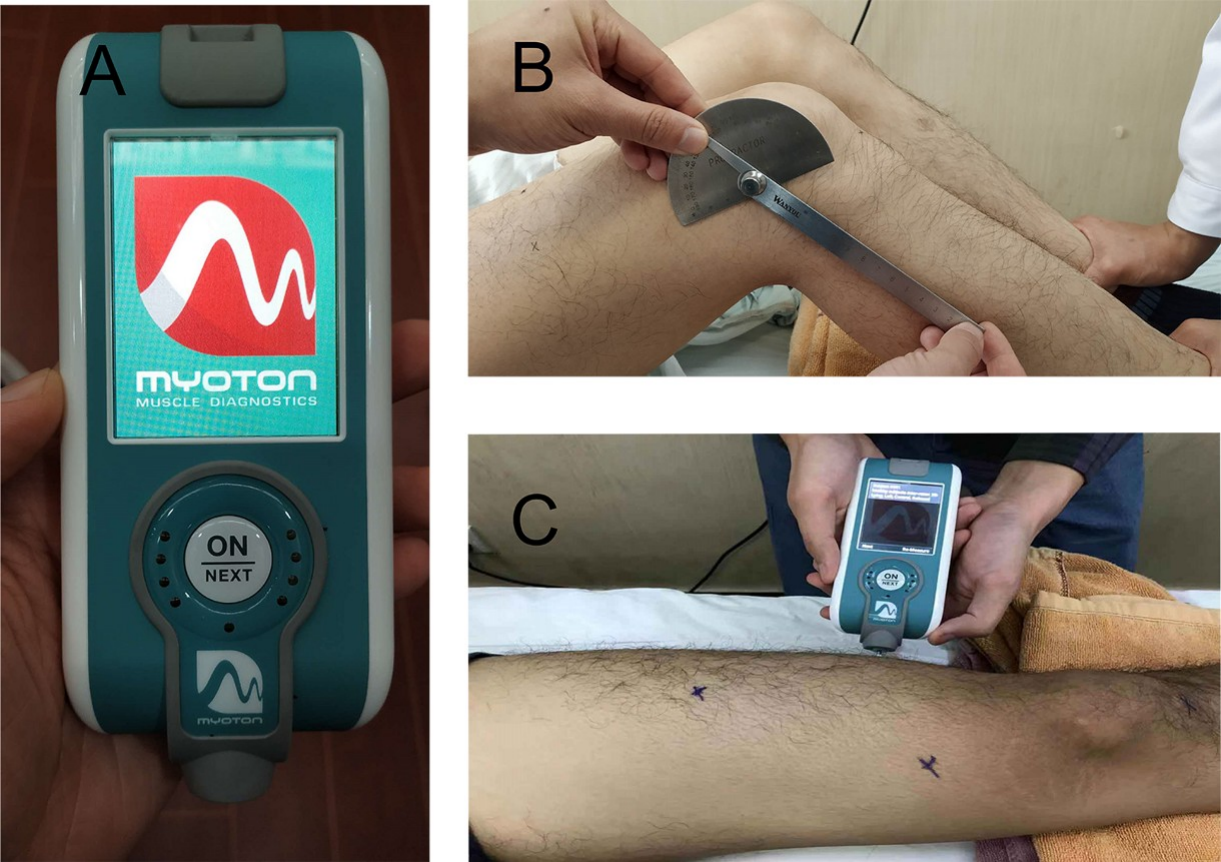
The MyotonPRO measurement technique. (A) MyotonPRO device; (B) Identification of measurement angle; (C) Measurement with MyotonPRO.

This single-center study was carried out at the orthopedics department of Guangdong Second Traditional Chinese Medicine Hospital. All the tests were carried out in the same room, where the room temperature was maintained around 25°C. The participants, who rested for 10 min before the test, were requested to remain in supine and resting positions on the examination couch. The measurement sites were marked with the knee and hip extended. The RF was measured at two-thirds of the distance between the anterior superior iliac spine (ASIS) and the superior pole of the patella[13]. To identify the muscle belly of VM and VL, subjects were requested to actively contract the lower limbs and force the hip and knee to extend; then, the measurement points were taken to be the most salient points of each belly (near the knee joint). The PT was measured at a point midway between the patella distal and the tuberosity of tibial when the knee was flexed at 90°. All the locations were marked by one operator (GqC), the measuring points were marked by a pen, and MyotonPRO was placed at these measuring points such that the probe was perpendicular to the skin surface of the tested muscle belly and tendon. The measurements at four sites were performed at 0°, 30°, 60°, and 90° of flexion of the knees. The hip flexed with knee position to make sure the lower limbs in the neutral position. A steel goniometer was used to quantify the angle of knee flexion (Fig1B). The measurements were performed bilaterally. The order was as follows: left RT, right RF; left VM; right VM; left VL; right VL; left PT; right PT. The measurement technique of MyotonPRO is illustrated in Fig 1A. The measurements were performed on each subject at different angles by one operator (GqC) based on the above-mentioned protocol, while another operator (JtW) maintained the lower limbs in a neutral position (Fig1B and 1C), and then, vice versa, with a 30 min-interval between two measurements on the same day. After a 7-day interval, the same subject was reassessed by the operator GqC, with JtW as the assistant.

### Recruitment

We used social media, the internal hospital network, and posters to recruit healthy participants from local university staff or students who were on clinical rotation in local hospitals. Participants who were interested in this study were required to communicate with one member of our team. All potential participants were required to fill out a basic questionnaire. Then, two members of our team (JtW, WX) screened the eligible participant. Any disagreement was arbitrated by a senior member (GcC).

### Sample population

Healthy young adults were included if (i) they were aged between 20 to 30 years; (ii) they did not have any musculoskeletal dysfunction; (iii) or systemic diseases; (iv) they did not perform intense exercises or play sports weekly.

The exclusion criteria were as follows: (i) those with a body mass index (BMI) ≥ 28 kg/m^2^ or ≤ 16 kg/m^2^; (ii) those with a history of fracture or surgery in lower limbs; (iii) pregnant or menstruating women; (iv) those who performed intense exercises, such as running for more than half an hour, or played sports, such as basketball or football, weekly; (v) those who were taking medication that could affect the musculoskeletal function; (vi) those who could not complete the entire process.

### Ethics

The Institutional Ethics Committee of Guangdong Second Traditional Chinese Medicine Hospital approved the study protocol (Approval No.: 2018(43)). All participants who met the criteria were recruited to engage in the study. Before the experiments, all participants were given time to consider whether they were interested in this study. If they agreed to participate in the trial, their written informed consent was obtained prior to data collection. All participants were free to withdraw from the trial at any stage. The study was registered with the Chinese Clinical Trial Registry (ChiCTR1800019407 on November 10, 2018).

### Equipment

A non-invasive handheld machine (MyotonPRO, Estonia) was used to collect tone and stiffness data of the muscles and tendons around the knee joint. First, the probe of MyotonPRO was held perpendicular to the skin surface. Then, it was pushed against the tested area to reach the required depth. After the red light turned green, five short impulses (tap interval was 0.8 s) were implemented automatically by the device in order to induce mechanical oscillations in the soft tissues. The MyotonPRO device provides data on the oscillation frequency (F, Hz) and dynamic stiffness (S, N/m) that can indicate the tone and stiffness, respectively. The mean values for tone and stiffness were calculated from the responses to the five impulses delivered.

### Parameters measured

The parameters of tone and stiffness were recorded by MyotonPRO. The device collects data through mechanical oscillations of soft tissues induced by mechanical impulses. The muscle tone is calculated as the oscillation frequency (Hz) = 1/T, where T represents the duration of oscillation. The stiffness value is calculated as the maximum acceleration of the oscillation and the deformation of the tissue detected by the transducer (N/m).

### Statistical analysis

We used SPSS 22 software (IBM, US) to conduct the data analysis. Demographic characteristics of the participants including age, gender, height, weight, and BMI were assessed by descriptive statistics. The normality distribution was assessed by the Kolmogorov–Smirnov test and frequency histograms. The intraclass correlation coefficient (ICC) was used to assess the inter- and intra-operator reliability. The model ICC (3,1) (two-way mixed model, single measures) and model ICC (2,2) (two-way random model, mean measures) were used to examine the intra-operator (measurements taken on two occasions separated by 7 days) and inter-operator (measurements by two operators) reliability, respectively. The reliability was considered excellent when the values of ICC exceeded 0.75. When the ICC range from 0.4 to 0.74, the reliability was considered to be good to fair, and when the value was less than 0.40, it was considered poor[25]. The absolute reliability was assessed using standard error of measurement (SEM, SEM = standard deviation×√1-ICC), minimal detectable change (MDC, MDC = 1.96×SEM×√2), and 95% limits of agreement (LOA). Bland–Altman plots obtained by the MedCalc 18 software (Software bvba, Ostend, Belgium) were used to assess inter- and intra-operator reliability to visualize the degree of agreement and to identify systematic bias. One-way ANOVA was used to compare the mean tone and stiffness (three measurements) index of the quadriceps femoris and PT among different angles, followed by post-hoc analysis with Bonferroni adjustment. The level of significance was determined as *p < 0*.*05*, and data were presented as mean ±SD.

## Results

### Demographics

Thirty healthy participants (15 females and 15 males, aged 24.7±1.6 years) were recruited in this study. Table 1 shows the characteristics of the enrolled participants. The data of height, weight, and BMI were 165.3±8.4 cm, 57.9±10.9 kg, and 21.0±2.8 kg/m^2^, respectively. Average mean values of normal data and mean values of three measurements are shown in Tables 2 and 3.

**Table 1:** A summary of the demographics of all participants.

Basic information	
Age (mean ± SD) years	24.7±1.6
Height (mean ± SD) cm	165.3±8.4
Weight (mean ± SD) kg	57.9±10.9
Body Mass Index (mean ± SD) kg/m^2^	21.0±2.8
Gender, Female/Male	15/15
Dominant Side, Left/Right	1/29

**Table 2:** Mean values ± standard deviation for measurement of tone and stiffness of quadriceps femoris and patellar tendon at each angle recorded by MyotonPRO.

Location	Angles Of knee	Variable	RF Mean ± SD			VM Mean ± SD			VL Mean ± SD			PT Mean ± SD		
			Operator1	Operator2	Operator1 (7days later)	Operator1	Operator2	Operator1 (7days later)	Operator1	Operator2	Operator1 (7days later)	Operator1	Operator2	Operator1 (7days later)
Dominant leg	0°	Frequency (Hz)	14.9±1.2	14.4±1.1	14.9±1.2	12.5±1.2	12.3±1.2	12.7±1.2	14.0±1.1	13.9±1.2	14.0±1.0	14.4±0.9	14.4±0.9	14.5±0.8
		Stiffness (N/m)	268.5±29.2	260.2±24.9	265.8±29.1	191.6±44.9	190.1±47.3	202.0±44.4	249.1±25.9	251.9±28.1	249.6±24.3	220.8±35.4	224.1±30.9	225.5±30.7
	30°	Frequency (Hz)	14.5±1.0	14.1±0.9	14.3±1.0	12.3±1.0	12.2±0.8	12.4±1.1	13.9±0.9	13.9±0.9	13.8±0.8	17.0±1.7	16.6±1.5	16.7±1.5
		Stiffness (N/m)	261±25.2	257.5±24.3	259.6±28.4	197.9±37.3	196.2±32.8	205.6±34.8	249.3±22.5	252.8±20.2	248.5±21.9	395.2±93.3	378.7±85.1	382.5±89
	60°	Frequency (Hz)	14.9±1.2	14.8±1	14.9±1.1	13.7±0.9	13.8±1.0	13.6±0.9	15.3±1.1	15.2±0.9	15.4±1.1	20.5±2.5	20.9±2.1	20.7±1.8
		Stiffness (N/m)	272.4±27.9	272±25.7	272.8±28.5	247.1±31	249.8±32.1	245.6±29.4	293±26.1	295.3±27.6	294.3±27.8	594.9±115.8	617.9±95.9	603.4±94.4
	90°	Frequency (Hz)	15.0±1.2	14.9±1.2	15.1±1.3	15.3±1.3	15.6±1.4	15.1±1.3	16.2±1.2	16.4±1.1	16.5±1.3	23.6±2.7	23.9±2.8	23.7±2.1
		Stiffness (N/m)	275.3±27.0	278.2±29.3	278.1±29.9	291.9±38.7	299.9±39.6	288.7±37.7	320.8±29.9	330±27.2	332.3±30.4	689.2±109.4	691.6±109.1	689.6±103.8
Non Dominant leg	0°	Frequency (Hz)	15±1.2	14.5±1.2	14.9±1.1	12.2±1.1	12±0.9	12.2±1.1	14.2±1.2	14.0±1.2	14.3±1.1	14.5±1.3	14.3±1.0	14.4±1.0
		Stiffness (N/m)	269.1±26.6	259.7±29.3	267.1±30.3	185.4±38.8	183.8±39.6	187.5±38.4	251.4±27	248.6±25.5	253.4±24.8	223.1±48.3	220±40.4	218.6±37.5
	30°	Frequency (Hz)	14.5±1.0	14.1±0.9	14.4±1.0	12.0±0.9	12.0±0.7	12.1±1.0	13.9±0.9	13.7±0.9	14±1.0	17.0±1.9	16.9±1.9	16.7±1.8
		Stiffness (N/m)	259.3±24.5	254.3±25	260.6±29.7	186.6±35.9	191.6±30.1	195.4±34.8	250.1±25.8	247.2±24.1	251.8±22.0	388.9±96.6	386±99.3	372±92.6
	60°	Frequency (Hz)	15±1.2	14.6±0.9	15.1±1.3	13.7±1.3	13.8±0.9	13.7±1.1	15.2±0.9	15.3±0.8	15.3±1.1	21.0±3.4	21.4±2.7	21.3±2.7
		Stiffness (N/m)	272.7±28.9	268.1±25.4	274.8±29.8	243.1±39.4	248.6±33.4	245.8±35.1	293.5±27.1	295.5±25.1	296.9±29.1	603.8±146.8	631.6±120.6	619.2±122.6
	90°	Frequency (Hz)	15.2±1.4	14.9±1.1	15.4±1.3	15.2±1.4	15.4±1.4	15.5±1.6	16.3±1.2	16.4±1.1	16.3±1.2	24.3±3.5	24.5±3.2	24.6±3.4
		Stiffness (N/m)	276.8±33.5	275.6±29.9	283.6±32.8	289.8±45.2	296.3±45.6	296.4±48.3	330.2±34.8	330.2±29.5	330.1±33.7	713.5±127.3	720.3±125.9	714.7±132.4

SD = standard deviation, RF = rectus femoris, VM = vastus medialis, VL = vastus lateralis, PT = patellar tendon; Operator1 means recorded by GcC, Operator2 means recorded by JtW.

**Table 3:** Mean values of three measurements and the difference at each angle.

Location	Angles Of knee	Variable	RF	VM	VL	PT
			Mean value of Three measurements	Mean value of Three measurements	Mean value of Three measurements	Mean value of Three measurements
Dominant leg	0°	Frequency (Hz)	14.8	12.1	14.2	14.4
		Stiffness (N/m)	265.3	185.6	251.1	220.6
	30°	Frequency (Hz)	14.3	12.1	13.9	16.8#
		Stiffness (N/m)	258.1	191.2	249.7	382.3#
	60°	Frequency (Hz)	14.9	13.7*	15.3*	21.2#*
		Stiffness (N/m)	271.9	245.8*	295.3*	618.2#*
	90°	Frequency (Hz)	15.2	15.4*Δ	16.3*Δ	24.5#*Δ
		Stiffness (N/m)	278.7	294.2*Δ	330.2*Δ	716.2#*Δ
	*P* value	Frequency	0.186	0.00	0.00	0.00
		Stiffness	0.027	0.00	0.00	0.00
Non Dominant leg	0°	Frequency (Hz)	14.8	12.5	14.0	14.4
		Stiffness (N/m)	264.8	194.6	250.2	223.5
	30°	Frequency (Hz)	14.3	12.3	13.9	16.8#
		Stiffness (N/m)	259.4	199.9	250.2	385.4#
	60°	Frequency (Hz)	14.9	13.7*	15.3*	20.7#*
		Stiffness (N/m)	272.4	247.5*	294.2*	605.4#*
	90°	Frequency (Hz)	15.2	15.3*Δ	16.4*Δ	23.7#*Δ
		Stiffness (N/m)	277.2*	293.5*Δ	327.7*Δ	690.1#*Δ
	*P* value	Frequency	0.067	0.00	0.00	0.00
		Stiffness	0.045	0.00	0.00	0.00

RF = Rectus Femoris, VM = Vastus Medialis, VL = Vastus Lateralis; PT = Patellar Tendon.

^*#*^*P<0*.*05* compare to 0°,

*P<0.*05* compare to 30°,

^Δ^ *P<0*.*05* compare to 60°.

### Difference in tone and stiffness of tissues at each angle

Differences in tone and stiffness of tissues at each angle are shown in Table 3. The one-way ANOVA and post-hoc analysis with Bonferroni adjustment indicated that there was no significant difference in the tone of RF between the different angles (P>0.05). In contrast, the tone and stiffness of PT showed significant differences among all angles (p<0.01). There also was no significant difference in measurements of tone and stiffness of VM and VL made at 0° compared to 30°(P > 0.05).

### Inter- and intra-operator reliability

MyotonPRO showed good to excellent inter- and intra-operator reliability between the sets of five repetitions shown in Tables 4 and 5. The within-day inter-operator reliability was excellent for muscles and tendons at different angles, regardless of the tone and stiffness (ICC 2,2 >0.78). More specifically, the ICC values of inter-operator reliability of RF ranged from 0.87 to 0.98, those of VM ranged from 0.79 to 0.98, those of VL ranged from 0.81 to 0.98, and those of PT ranged from 0.78 to 0.97. For the between-day intra-operator reliability, the mean of a single set of five taps was good to excellent (ICC 3,1 = 0.41–0.90) with wide 95% confidence intervals. More specifically, the ICC values of intra-operator reliability of RF ranged from 0.70 to 0.87, those of VM ranged from 0.53 to 0.90, those of VL ranged from 0.41 to 0.90, and those of PT ranged from 0.51 to 0.90. The overall mean values of inter-operator ICC (95%CI) was 0.87 (0.82–0.97), and the overall mean values of intra-operator ICC (95%CI) was 0.74 (0.56–0.86).

**Table 4:** The results of Inter-operator ICC values and 95% CI.

Location	Angles Of knee	Variable	RF	VM	VL	PT
			ICC (95% CI)	ICC (95% CI)	ICC (95% CI)	ICC (95% CI)
Dominant leg	0°	Frequency (Hz)	0.90(0.48–0.96)	0.98(0.93–0.99)	0.98(0.95–0.99)	0.92(0.83–0.96)
		Stiffness (N/m)	0.92(0.75–0.97)	0.98(0.97–0.99)	0.97(0.94–0.99)	0.93(0.85–0.97)
	30°	Frequency (Hz)	0.89(0.66–0.95)	0.96(0.91–0.98)	0.92(0.84–0.96)	0.87(0.73–0.94)
		Stiffness (N/m)	0.94(0.87–0.97)	0.97(0.93–0.98)	0.92(0.82–0.96)	0.89(0.76–0.95)
	60°	Frequency (Hz)	0.96(0.92–0.98)	0.86(0.71–0.94)	0.85(0.69–0.93)	0.90(0.79–0.95)
		Stiffness (N/m)	0.98(0.95–0.99)	0.90(0.79–0.95)	0.89(0.76–0.95)	0.92(0.81–0.96)
	90°	Frequency (Hz)	0.95(0.90–0.98)	0.93(0.85–0.97)	0.89(0.78–0.95)	0.90(0.79–0.95)
		Stiffness (N/m)	0.97(0.94–0.99)	0.92(0.83–0.96)	0.84(0.64–0.93)	0.97(0.93–0.99)
Non Dominant leg	0°	Frequency (Hz)	0.90(0.69–0.96)	0.96(0.91–0.98)	0.99(0.97–0.99)	0.90(0.79–0.95)
		Stiffness (N/m)	0.91(0.70–0.97)	0.97(0.93–0.98)	0.97(0.94–0.99)	0.78(0.53–0.89)
	30°	Frequency (Hz)	0.90(0.40–0.97)	0.90(0.79–0.95)	0.81(0.61–0.91)	0.94(0.87–0.97)
		Stiffness (N/m)	0.94(0.86–0.97)	0.94(0.86–0.97)	0.94(0.88–0.97)	0.96(0.91–0.98)
	60°	Frequency (Hz)	0.87(0.54–0.95)	0.79(0.56–0.90)	0.90(0.78–0.95)	0.92(0.83–0.96)
		Stiffness (N/m)	0.96(0.90–0.98)	0.89(0.76–0.95)	0.88(0.75–0.94)	0.92(0.83–0.96)
	90°	Frequency (Hz)	0.94(0.83–0.98)	0.95(0.90–0.98)	0.97(0.93–0.98)	0.95(0.90–0.98)
		Stiffness (N/m)	0.98(0.96–0.99)	0.95(0.90–0.98)	0.95(0.90–0.98)	0.97(0.94–0.99)

ICC = Intraclass Correlation Coefficients, CI = Confidence Intervals, RF = Rectus Femoris, VM = Vastus Medialis, VL = Vastus Lateralis; PT = Patellar Tendon

**Table 5:** The results of Intra-operator ICC values and 95% CI.

Location	Angles Of knee	Variable	RF	VM	VL	PT
			ICC (95% CI)	ICC (95% CI)	ICC (95% CI)	ICC (95% CI)
Dominant leg	0°	Frequency (Hz)	0.72(0.48–0.86)	0.90(0.80–0.95)	0.87(0.75–0.94)	0.64(0.37–0.81)
		Stiffness (N/m)	0.81(0.63–0.90)	0.87(0.70–0.94)	0.90(0.81–0.95)	0.51(0.18–0.73)
	30°	Frequency (Hz)	0.75(0.54–0.87)	0.84(0.70–0.92)	0.72(0.49–0.86)	0.64(0.38–0.81)
		Stiffness (N/m)	0.76(0.56–0.88)	0.60(0.32–0.79)	0.49(0.16–0.72)	0.74(0.52–0.87)
	60°	Frequency (Hz)	0.84(0.69–0.92)	0.70(0.46–0.84)	0.83(0.67–0.92)	0.70(0.46–0.85)
		Stiffness (N/m)	0.87(0.73–0.93)	0.58(0.28–0.78)	0.69(0.44–0.84)	0.78(0.58–0.89)
	90°	Frequency (Hz)	0.83(0.67–0.92)	0.76(0.55–0.88)	0.84(0.65–0.93)	0.73(0.51–0.86)
		Stiffness (N/m)	0.82(0.65–0.91)	0.58(0.29–0.78)	0.64(0.34–0.81)	0.80(0.63–0.90)
Non Dominant leg	0°	Frequency (Hz)	0.78(0.58–0.89)	0.81(0.64–0.91)	0.58(0.30–0.78)	0.62(0.34–0.80)
		Stiffness (N/m)	0.70(0.46–0.85)	0.86(0.73–0.93)	0.79(0.65–0.90)	0.59(0.30–0.78)
	30°	Frequency (Hz)	0.74(0.53–0.87)	0.76(0.56–0.88)	0.70(0.45–0.84)	0.82(0.66–0.91)
		Stiffness (N/m)	0.70(0.45–0.84)	0.53(0.23–0.75)	0.41(0.06–0.67)	0.80(0.62–0.90)
	60°	Frequency (Hz)	0.74(0.51–0.87)	0.78(0.59–0.89)	0.70(0.47–0.85)	0.83(0.67–0.91)
		Stiffness (N/m)	0.77(0.58–0.89)	0.55(0.24–0.76)	0.42(0.07–0.68)	0.85(0.71–0.93)
	90°	Frequency (Hz)	0.80(0.77–0.95)	0.84(0.68–0.92)	0.82(0.65–0.91)	0.87(0.75–0.94)
		Stiffness (N/m)	0.85(0.69–0.93)	0.60(0.31–0.79)	0.69(0.43–0.84)	0.90(0.81–0.95)

ICC = Intraclass Correlation Coefficients, CI = Confidence Intervals, RF = Rectus Femoris, VM = Vastus Medialis, VL = Vastus Lateralis; PT = Patellar Tendon

Tables 6 and 7 show the SEM, MDC, and 95% LOA. The SEM for all inter-operator tissue tone measurements were below 1 Hz (0.1–0.9 Hz). The SEM for all inter-operator tissue stiffness measurements ranged from 3.8 to 37.9 N/m. The SEM for all intra-operator tissue tone measurements ranged from 0.5 to 1.3 Hz, while intra-operator tissue stiffness measurements ranged from 7.9 to 52.0 N/m. For the tone of pooled tissues, the SEM for inter-operator ranged from 0.3 to 0.6 Hz and the SEM for intra-operator ranged from 0.3 to 1.5Hz.

**Table 6:** The results of Inter-operator SEM, MDC and 95% LOA.

Location	Angles Of knee	Variable	RF					VM					VL					PT				
			mean	SEM	MDC	95% LOA		mean	SEM	MDC	95% LOA		mean	SEM	MDC	95% LOA		mean	SEM	MDC	95% LOA	
						Lower	Upper				Lower	Upper				Lower	Upper				Lower	Upper
Dominant leg	0°	Frequency (Hz)	14.7	0.4	1.1	-0.6	1.6	12.4	0.2	0.6	-0.5	0.8	14.0	0.2	0.6	-0.6	0.7	14.4	0.3	0.8	-1.0	1.0
		Stiffness (N/m)	264.3	7.7	21.3	-16.1	32.6	190.8	6.5	18.0	-21.3	24.3	250.5	4.6	12.8	-19.7	14.0	222.4	8.7	24.1	-36.7	30.1
	30°	Frequency (Hz)	14.3	0.3	0.8	-0.7	1.4	12.2	0.2	0.6	-0.7	0.8	13.9	0.3	0.8	-1.0	1.0	16.8	0.6	1.7	-1.7	2.4
		Stiffness (N/m)	259.3	6.0	16.6	-19.2	26.2	197.1	6.0	16.6	-24.1	27.3	251.1	7.0	19.4	-26.5	19.5	386.9	29.5	81.8	-93.3	126.2
	60°	Frequency (Hz)	14.8	0.2	0.6	-0.7	0.9	13.8	0.4	1.1	-1.4	1.2	15.3	0.4	1.1	-1.3	1.6	20.7	0.7	1.9	-3.1	2.3
		Stiffness (N/m)	272.2	3.8	10.5	-15.4	16.3	248.4	9.9	27.4	-40.1	34.7	294.2	8.8	24.4	-49.8	44.7	606.4	30	83.2	-133.9	87.9
	90°	Frequency (Hz)	15.0	0.3	0.8	-0.9	1.0	15.5	0.4	1.1	-1.5	1.1	16.3	0.4	1.1	-1.6	1.2	23.7	0.9	2.5	-2.7	2.2
		Stiffness (N/m)	276.8	4.8	13.3	-20.3	14.5	295.9	11.0	30.5	-47.2	31.1	325.4	11.5	31.9	-48.4	30.0	690.4	18.8	52.1	-78.3	73.4
Non Dominant leg	0°	Frequency (Hz)	14.7	0.4	1.1	-0.2	1.6	12.1	0.2	0.6	-0.6	0.9	14.1	0.1	0.3	-0.3	0.8	14.4	0.4	1.1	-1.2	1.5
		Stiffness (N/m)	264.4	8.4	23.3	-16.3	35.1	184.6	6.7	18.6	-26.0	29.2	250.0	4.5	12.5	-13.7	19.4	221.5	20.7	57.4	-72.1	78.2
	30°	Frequency (Hz)	14.3	0.3	0.8	-0.4	1.3	12.0	0.3	0.8	-1.0	1.0	13.8	0.4	1.1	-1.3	1.6	16.9	0.5	1.4	-1.7	2.0
		Stiffness (N/m)	256.8	6.0	16.6	-16.4	26.3	189.1	8.1	22.5	-36.3	26.3	248.7	6.1	16.9	-19.6	25.4	387.4	19.4	53.8	-78.0	83.8
	60°	Frequency (Hz)	14.8	0.4	1.1	-0.7	1.6	13.8	0.5	1.4	-1.9	1.7	15.2	0.3	0.8	-1.1	1.0	21.2	0.9	2.5	-3.7	2.8
		Stiffness (N/m)	270.4	5.4	15	-16.0	25.3	245.8	12.0	33.3	-50.8	39.9	294.5	9.0	24.9	-35.7	31.7	617.7	37.9	105.1	-162.3	106.7
	90°	Frequency (Hz)	15.1	0.3	0.8	-0.7	1.3	15.3	0.3	0.8	-1.3	1.0	16.4	0.2	0.6	-0.9	0.7	24.4	0.7	1.9	-3.1	2.7
		Stiffness (N/m)	276.2	4.5	12.5	-15.5	18.0	293.1	10.1	28	-42.4	29.5	330.2	7.1	19.7	-26.9	26.9	716.9	21.8	60.4	-89.6	76.0

SEM = Standard Error of Measurements, MDC = Minimal Detectable Change, LOA = Limits of Agreement, RF = Rectus Femoris, VM = Vastus Medialis, VL = Vastus Lateralis, PT = Patellar Ten

**Table 7:** The results of Intra-operator SEM, MDC and 95% LOA.

Location	Angles Of knee	Variable	RF					VM					VL					PT				
			mean	SEM	MDC	95% LOA		mean	SEM	MDC	95% LOA		mean	SEM	MDC	95% LOA		mean	SEM	MDC	95% LOA	
						Lower	Upper				Lower	Upper				Lower	Upper				Lower	Upper
Dominant leg	0°	Frequency (Hz)	14.9	0.6	1.7	-1.8	1.9	12.6	0.4	1.1	-1.2	0.9	14.0	0.4	1.1	-1.0	1.0	14.4	0.5	1.4	-1.6	1.4
		Stiffness (N/m)	267.2	12.6	34.9	-33.1	38.4	196.8	16.1	44.6	-52.1	31.3	249.4	7.9	21.9	-22.4	21.4	223.2	23.1	64	-69.5	60.1
	30°	Frequency (Hz)	14.4	0.5	1.4	-1.2	1.6	12.3	0.4	1.1	-1.2	1.0	13.9	0.4	1.1	-1.3	1.4	16.8	1	2.8	-2.2	3.0
		Stiffness (N/m)	260.3	13.0	36.0	-35.1	38.0	201.8	22.8	63.2	-48.6	33.0	248.9	12.3	34.1	-24.2	25.8	388.8	46.2	128.1	-116.4	141.8
	60°	Frequency (Hz)	14.9	0.5	1.4	-1.3	1.2	13.7	0.5	1.4	-1.2	1.5	15.4	0.4	1.1	-1.3	1.3	20.6	1.2	3.3	-3.4	3.2
		Stiffness (N/m)	272.6	10.1	28	-30.0	29.2	246.3	19.4	53.8	-33.7	36.6	293.6	14.9	41.3	-38.4	35.7	599.1	49.2	136.4	-148.4	131.3
	90°	Frequency (Hz)	15.0	0.5	1.4	-1.5	1.3	15.2	0.6	1.7	-1.5	2.0	16.3	0.5	1.4	-1.6	1.0	23.7	1.2	3.3	-3.6	3.3
		Stiffness (N/m)	276.7	12.0	33.3	-36.8	31.2	290.3	24.6	68.2	-48.2	54.7	326.5	18.3	50.7	-48.9	26.0	689.4	47.3	131.1	-133.3	132.5
Non Dominant leg	0°	Frequency (Hz)	14.9	0.5	1.4	-1.4	1.6	12.2	0.5	1.4	-1.3	1.3	14.2	0.8	2.2	-2.3	2.0	14.4	0.7	1.9	-1.9	2.0
		Stiffness (N/m)	268.1	15.5	43	-41.6	45.7	186.5	14.3	39.6	-42.1	37.9	252.4	11.8	32.7	-34.9	31.1	220.9	27.5	76.2	-72.9	81.7
	30°	Frequency (Hz)	14.5	0.5	1.4	-1.2	1.6	12.1	0.5	1.4	-1.4	1.2	13.9	0.5	1.4	-1.6	1.4	16.8	0.8	2.2	-1.8	2.4
		Stiffness (N/m)	260.0	14.8	41	-43.3	40.6	191.0	24.2	67.1	-45.2	27.6	251.0	18.3	50.7	-38.3	34.9	380.4	42.1	116.7	-99.5	133.2
	60°	Frequency (Hz)	15.0	0.6	1.7	-1.8	1.7	13.7	0.6	1.7	-1.6	1.6	15.3	0.6	1.7	-1.7	1.4	21.1	1.3	3.6	-3.8	3.2
		Stiffness (N/m)	273.8	14	38.8	-41.1	37.0	244.5	24.8	68.7	-43.7	38.3	295.2	21.3	59.0	-50.3	43.5	611.5	52.0	144.1	-159.3	128.5
	90°	Frequency (Hz)	15.3	0.6	1.7	-1.9	1.4	15.4	0.6	1.7	-1.9	1.4	16.3	0.5	1.4	-1.5	1.5	24.5	1.2	3.3	-3.8	3.1
		Stiffness (N/m)	280.2	12.8	35.5	-41.1	27.6	293.1	29.4	81.5	-58.7	45.6	330.1	18.9	52.4	-42.2	42.4	714.1	40.7	112.8	-114.9	112.4

SEM = Standard Error of Measurements, MDC = Minimal Detectable Change, LOA = Limits of Agreement, RF = Rectus Femoris, VM = Vastus Medialis, VL = Vastus Lateralis, PT = Patellar Tendon

The MDC for all inter-operator tissue tone measurements ranged between 0.3 to 2.5 Hz. The MDC for all inter-operator tissue stiffness measurements ranged between 10.5 to 105.1 N/m. The MDC for all intra-operator tissue tone measurements ranged from 1.1 to 3.3 Hz, while intra-operator tissue stiffness measurements ranged from 21.9 to 144.1 N/m.

**Fig 2:**
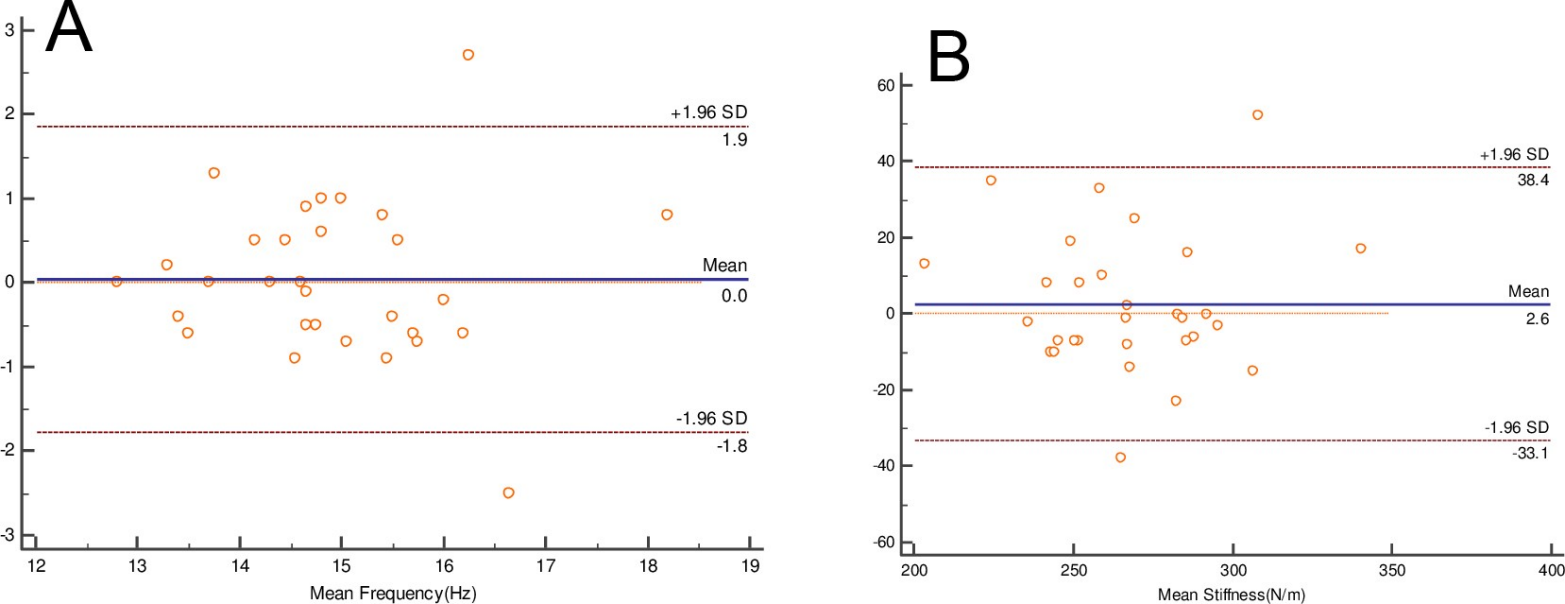
Examples Bland–Altman plots for MyotonPRO measurement of dominant rectus femoris tone(A) and stiffness(B) of healthy males. 95% limits of agreement and mean difference marked with dotted(––) and solid (—)lines.

The Bland–Altman analysis showed the level of inter- and intra-operator agreements, the result of which are presented in Tables 4 and 5. The 95% LOA values were wider for between-day results than within-day results. The plots showed little systematic bias between the two measurements (Fig 2). The 95% LOA values of PT were wider than those of other tissues at each level. The 95% LOA of pooled muscle tone on different knee angles for inter-operator and intra-operator were between −2.4 to 2.3 Hz and −2.8 to 2.7Hz, respectively.

## Discussion

The present study is, to date, the first to use MyotonPRO to measure tone and stiffness of quadriceps femoris (RF, VM, and VL) and PT at different angles of the knee joint in a healthy population. In this study, the portable MyotonPRO device showed good inter- and intra-operator reliability. The within-day inter-operator reliability was shown to be excellent for measuring muscles and tendons at all angles, and the between-day Intra-operator reliability was indicated to be good to excellent. There was no difference in RF measurements with variation in the angle, while there was a significant difference in PT measurements with a change in the angle.

### Within-day inter- and between-day intra-operator reliability

It has been reported that MyotonPRO is a reliable device to assess the mechanical properties of several muscles and tendons. However, studies published in the literature have not yet used MyotonPRO to measure the reliability of RF, VM, VL, and PT in healthy populations as well as at different positions of the knee joint.

ICC is a descriptive statistic that can be used to reflect on both degrees of consistency and agreement when quantitative measurements are performed [26]. The values of ICC range from 0 to 1; a high ICC value indicates close agreement between measurements [11]. Some authors consider that the validity of ICC is primarily based on inter-rater reliability data, and the device would be considered reliable if ICC values of intra-operator reliability are higher [27].

In terms of inter-operator reliability, the ICC values were observed to range from 0.78 to 0.98, i.e., they were greater than 0.75; this indicated that the inter-rater reliability was excellent at all parts for all angles. The within-day inter-operator ICC values in RF ranged between 0.90 to 92 (including dominant leg and non-dominant leg); these results were in accordance with those of a published study[24] where the within-day reliability of RF tone and stiffness at 0° was measured (ICC = 0.92 to 0.93). The ICC values (0.97 to 0.99) of within-day in a previous study [12] were higher than those of our study. This difference may be related to the individual measurement habits of operators. The former study considered the same operator, while ours considered two different operators.

Regarding intra-operator reliability, we observed that ICC values ranged from 0.41 to 0.90, i.e., they were greater than 0.4; this indicated that the intra-operator reliability was good to excellent at all parts for all angles. Our study showed that the between-day intra-operator ICC values of RF at 0° were between 0.70 to 0.81; these results are close to those of a study conducted in Southampton, UK (ICC = 0.81 to 0.83) [19]. The first reason for lower ICC values may be related to the choice of the ICC model; our study selected a single-measure model, while the previous study selected a mean measure model. Commonly, the values of ICC mean measure models are higher than those of ICC single measure models. The second reason may be related to the participants; in our study, the participants included both males and females while their study only considered males. A previous study on VL [22], indicated that the ICC value of VL stiffness measured using a myometer (Myoton-2) was 0.4, which was poorer than the result in our study. This difference may be associated with the measurement device. MyotonPRO is a novel device that consists of a triaxial accelerometer and a system to increase stability, which allows multidirectional measurements in relation to the gravity vector, while Myoton-2 consists of only a single accelerometer [24]. Moreover, a study [28] on PT tone and stiffness at 90° reported that the within-day ICC values for PT tone (ICC = 0.96) and stiffness (ICC = 0.96) were close to those of our study (tone ICC = 0.95, stiffness ICC = 0.97).

Regarding ICC values of different angles, to the best of our knowledge, data for comparison are limited in published articles. A previous study[23] investigated the reliability of computerized muscle tonometer measuring RF tone at different knee angles(0–60°), they found the ICC values ranged from 0.75 to 0.99. The ICC values in our study ranged from 0.87 to 0.96(0–60°). In our study, we observed that ICC values of PT and RF were higher at 90° than at 0°, regardless of inter-operator or intra-operator and tone or stiffness. A previous study suggested that subcutaneous fat may influence Myoton parameters [29]. According to its structure, the higher ICC values may be related to the subcutaneous fat within the knee [30]. When the knee joint is extended, the subcutaneous fat assembled can affect the measurements of slack PT as well as RF. As the angle reaches 90°, PT and RF remains at a strained status where the subcutaneous fat has little effect on the measurements. According to the ICC values, the best angle to measure PT and RF was 90°. For VM and VL, the best angle was 0°. However, our study only investigated four angles, and more angles should be conducted in the future.

Essentially, the within-day inter-operator reliability of MyotonPRO was better than the between-day intra-operator reliability. Furthermore, our study may provide a new method to increase the inter- and intra-operator reliability. This means that if the ICC values at the 0° position are poor, we may consider other angles of the knee joint. The reliability of MyotonPRO in various muscles and tendons need to be considered using more data, as the accuracy of this technique can be influenced by the architecture of tissues.

### SEM, MDC, and Bland–Altman analysis

The SEM and MDC values provide guidance for expected errors [31], indicating the real difference in tone and stiffness. SEM may be considered as the estimation of how repeated measures tend to be distributed around the “true” score. SRD value considers the minimum amount of change that could be interpreted as an actual change[25]. The smaller the values of SEM and MDC, the higher the reliability of the device. The Bland–Altman plots aim to identify systematic bias.

Few studies have been conducted in this regard and there is insufficient data for a comparison of SEM and MDC values of tissues. The SEM of tone observed in the present study was less than 1.3 Hz and 15.5 N/m for stiffness. For RF at 0°, our study indicated that the within-day SEM values ranged from 0.4 Hz to 8.4 Hz and the within-day MDC values ranged from 1.1 to 23.3 Hz. The results of within-day SEM and MDC values were consistent with the results of a previous study [19], where the within-day SEM values ranged from 0.49 to 10.4 Hz and the MDC values ranged from 1.34 to 28.8 Hz. The slightly higher between-day SEM and MDC values of their study may be related to the lower between-day reliability. SEM = standard deviation×√1-ICC. The smaller of ICC values, the higher the SEM; MDC = 1.96×SEM×√2, the lager the SEM, the higher the MDC. Previous studies have limited information on MyotonPRO when measuring VM, VL, and PT. In the present study, particularly with respect to the PT between-day intra-operator, the lowest value found for PT MDC values was 64 N/m and the highest value was 144.1 N/m. In this context, we advise researchers to consider MDC in future reliability studies of tone and stiffness.

The purpose of the Bland-Altman analysis is to identify systematic bias. The Bland–Altman plots indicated little systematic bias between the two measurements. Few studies have conducted Bland–Altman analyses so there is little scope for comparison with published studies. For between-day RF measurements, the results of the present study (mean frequency 0, 95% LOA ranged from -1.8 to 1.9; mean stiffness 2.6, 95% LOA ranged from -33.1 to 38.4) are consistent with those reported by Lucy Aird [12]. In their study, MyotonPRO was used to measure between-day frequency and stiffness and similar results were observed (mean frequency 0.17, 95% LOA ranged from -2.26 to 2.59; mean stiffness 2.21, 95% LOA ranged from -36.65 to 41.06). The slight difference may be related to the age and gender of the participants. In our study, the 95% LOA values were wider for between-day values than within-day values. This was also consistent with the results reported in Lucy Aird’s study.

### Muscles and tendon mechanical properties

The tone and stiffness of quadriceps femoris (RF, VM, and VL) and PT were quantified by MyotonPRO in healthy young participants at different knee joint angles (Table 3). For RF at 0°, the muscle tone ranged from 14.4 to 15.0 Hz. The range of stiffness was 260.2 to 269.1 N/m, which was similar to the values found using Myoton-2 for five females and five males with a mean stiffness of 268 N/m in the right quadriceps [22]. Our result was lower than that of a previous study on 21 young males (288 N/m) [24]. The lower values may be related to gender. The tone of RF was not changed by the knee angles, and there were differences in the stiffness at 30° and that at 90°. The results were contrary to those of a study where stiffness was measured by ultrasound shear wave elastography[32]. They showed that passive RF stiffness begins to increase when the knee angle at 45°. This difference may be related to RF architectural structure and measurement way. RF insertion is on the pelvis, and passive tension of RF would be affected by both hip and knee flexion. In our study, we measured the tone and stiffness of RF at different angles when the hip and knee both flexed (less stretch in our study). The higher tone and stiffness values of PT as knee angles increase are closely related to the anatomy of PT as well as subcutaneous fat, which cause PT strain when the knee is flexed. The changes in the tone and stiffness of PT were considerable with changes in knee positions. In the current study, we used the within-day mean tone and stiffness index to describe the tone of the PT. The means of the tone were 14.4 N at 0°, 16.9 N at 30°, 21.0 N at 60°, and 24.1 N at 90°, and the means of stiffness were 223.1 N/m at 0°, 387.2 N/m at 30°, 612.1 N/m at 60°, and 703.1 N/m at 90°; these results can provide a reference for future studies. Fiorella Celsi Young et al [33] used MyotonPRO to conduct measurements on healthy males (mean age: 27.3 years) in Santiago. The means of stiffness were 902 N/m for the dominant limb and 862 N/m for the non-dominant limb at 90°. The lower stiffness value may be associated with the race, gender, and knee position of the participants (our study adopted the supine position while theirs adopted the sitting position). To the best of our knowledge, studies have not been conducted on the tone and stiffness of muscles and tendons of participants of different races. This can be considered as a topic of future research.

### Limitations

The present results should be considered with several limitations. First, participants who had not performed any weekly strenuous exercise in the week before joining the study were considered. Although the participants were advised by our team to limit their activities before the experiments, this could not be accurately controlled. We can only ensure that the participants had sufficient time to rest before the experiments were conducted. Second, the sample size was too small, even if it reached the minimum requirement of numbers to examine the reliability (n = 20) [34]. Furthermore, all participants were of local hospitals and universities. This means that the enrolled participants were not generalizable. Third, sometimes it was difficult to locate the sites of the VM and VL in females, because they were not obvious. Measuring multiple sites and ways of relocating them reliably could be explored in future clinical research. Fourth, the MyotonPRO technology also has limitations, because it tests the properties of a certain muscle or tendon, while including the soft tissue above or below the muscle fiber or tendon. Therefore, the “true” values are not true. Previous studies have proved that the stiffness of Achilles's tendon and erector spinae measured by the myometer was consistent with those measured by ultrasound shear wave elastography. Future studies should focus on this topic. Fifth, the relaxation was not measured by electromyography; therefore, it was not possible to ensure that the lower limbs were in a resting state. To reduce the error, all the participants were measured by the two operators (GqC, JtW), and we reminded the participants to relax their lower limbs during the execution. Sixth, Inter-operator reliability may have been higher in the present study because one operator marked the location to be measured for both operators to use.

In conclusion, our study established the tone and stiffness values of quadriceps femoris (RF, VM, VL) and PT in healthy humans at different knee angles using MyotonPRO. The present study also suggested that the MyotonPRO is an acceptable device for the detection of tone and stiffness of quadriceps femoris and PT. The device will be useful for studying the mechanical properties of muscles and tendons, such as training, disease, growth, and aging.

## Supporting information

S1 Table A summary of the demographics of all participants. (PDF)

S2 Table Mean values ± standard deviation for measurement of tone and stiffness of quadriceps femoris and patellar tendon at each angle recorded by MyotonPRO. SD = standard deviation, RF = rectus femoris, VM = vastus medialis, VL = vastus lateralis, PT = patellar tendon; Operator1 means recorded by GcC, Operator2 means recorded by JtW.
(PDF)

S3 Table Mean value of three measurements and the difference at each angle. RF = Rectus Femoris, VM = Vastus Medialis, VL = Vastus Lateralis; PT = Patellar Tendon. ^*#*^*P<0*.*05* compare to 0°, *P<0.*05* compare to 30°, ^Δ^ *P<0*.*05* compare to 60°.
(PDF)

S4 Table The results of inter-operator ICC values and 95% CI. ICC = Intraclass Correlation Coefficients, CI = Confidence Intervals, RF = Rectus Femoris, VM = Vastus Medialis, VL = Vastus Lateralis; PT = Patellar Tendon
(PDF)

S5 Table The results of intra-operator ICC values and 95% CI. ICC = Intraclass Correlation Coefficients, CI = Confidence Intervals, RF = Rectus Femoris, VM = Vastus Medialis, VL = Vastus Lateralis; PT = Patellar Tendon
(PDF)

S6 Table The results of inter-operator SEM, MDC and 95% LOA. SEM = Standard Error of Measurements, MDC = Minimal Detectable Change, LOA = Limits of Agreement, RF = Rectus Femoris, VM = Vastus Medialis, VL = Vastus Lateralis, PT = Patellar Tendon
(PDF)

S7 Table The results of intra-operator SEM, MDC and 95% LOA. SEM = Standard Error of Measurements, MDC = Minimal Detectable Change, LOA = Limits of Agreement, RF = Rectus Femoris, VM = Vastus Medialis, VL = Vastus Lateralis, PT = Patellar Tendon
(PDF)

S1 Fig The MyotonPRO measurement technique. (A) MyotonPRO device; (B) Identification of measurement angle; (C) Measurement with MyotonPRO.
(PDF)

S2 Fig Examples Bland–Altman plots for MyotonPRO measurement of dominant rectus femoris tone(A) and stiffness(B) of healthy males. 95% limits of agreement and mean difference marked with dotted(––) and solid (—)lines.
(PDF)

S1 Data RF = Rectus Femoris, VM = Vastus Medialis, VL = Vastus Lateralis, PT = Patellar Tendon. (XLSX)
