# Supplementary material for: Reliability of a portable device for quantifying tone and stiffness of quadriceps femoris and patellar tendon at different knee flexion angles
Source: PLoS One. 2019 Jul 31;14(7):e0220521. doi: 10.1371/journal.pone.0220521 (PMC6668831; doi:10.1371/journal.pone.0220521)
Supplement: S1 Table — (PDF) [file pone.0220521.s001.pdf]

S1 Table. A summary of the demographics of all participants.

Basic information	
Age (mean ± SD) years	24.7±1.6
Height (mean ± SD) cm	165.3±8.4
Weight (mean ± SD) kg	57.9±10.9
Body Mass Index (mean ± SD) kg/m^2	21.0±2.8
Gender, Female/Male	15/15
Dominant Side, Left/Right	1/29
