# Supplementary material for: Reliability of a portable device for quantifying tone and stiffness of quadriceps femoris and patellar tendon at different knee flexion angles
Source: PLoS One. 2019 Jul 31;14(7):e0220521. doi: 10.1371/journal.pone.0220521 (PMC6668831; doi:10.1371/journal.pone.0220521)
Supplement: S2 Table — SD = standard deviation, RF = rectus femoris, VM = vastus medialis, VL = vastus lateralis, PT = patellar tendon; Operator1 means recorded by GcC, Operator2 means recorded by JtW. (PDF) [file pone.0220521.s002.pdf]

Table 2. Mean values ± standard deviation for measurement of tone and stiffness of quadriceps femoris and patellar tendon at each angle recorded by MyotonPRO

Location	Angles Of knee	Variable	RF Mean ± SD			VM Mean ± SD			VL Mean ± SD			PT Mean ± SD		
			Operator1	Operator2	Operator1 (7days later)	Operator1	Operator2	Operator1 (7days later)	Operator1	Operator2	Operator1 (7days later)	Operator1	Operator2	Operator1 (7days later)
Dominant leg	0°	Frequency (Hz)	14.9±1.2	14.4±1.1	14.9±1.2	12.5±1.2	12.3±1.2	12.7±1.2	14.0±1.1	13.9±1.2	14.0±1.0	14.4±0.9	14.4±0.9	14.5±0.8
		Stiffness (N/m)	268.5±29.2	260.2±24.9	265.8±29.1	191.6±44.9	190.1±47.3	202.0±44.4	249.1±25.9	251.9±28.1	249.6±24.3	220.8±35.4	224.1±30.9	225.5±30.7
	30°	Frequency (Hz)	14.5±1.0	14.1±0.9	14.3±1.0	12.3±1.0	12.2±0.8	12.4±1.1	13.9±0.9	13.9±0.9	13.8±0.8	17.0±1.7	16.6±1.5	16.7±1.5
		Stiffness (N/m)	261±25.2	257.5±24.3	259.6±28.4	197.9±37.3	196.2±32.8	205.6±34.8	249.3±22.5	252.8±20.2	248.5±21.9	395.2±93.3	378.7±85.1	382.5±89
	60°	Frequency (Hz)	14.9±1.2	14.8±1	14.9±1.1	13.7±0.9	13.8±1.0	13.6±0.9	15.3±1.1	15.2±0.9	15.4±1.1	20.5±2.5	20.9±2.1	20.7±1.8
		Stiffness (N/m)	272.4±27.9	272±25.7	272.8±28.5	247.1±31	249.8±32.1	245.6±29.4	293±26.1	295.3±27.6	294.3±27.8	594.9±115.8	617.9±95.9	603.4±94.4
	90°	Frequency (Hz)	15.0±1.2	14.9±1.2	15.1±1.3	15.3±1.3	15.6±1.4	15.1±1.3	16.2±1.2	16.4±1.1	16.5±1.3	23.6±2.7	23.9±2.8	23.7±2.1
		Stiffness (N/m)	275.3±27.0	278.2±29.3	278.1±29.9	291.9±38.7	299.9±39.6	288.7±37.7	320.8±29.9	330±27.2	332.3±30.4	689.2±109.4	691.6±109.1	689.6±103.8
Non Dominant leg	0°	Frequency (Hz)	15±1.2	14.5±1.2	14.9±1.1	12.2±1.1	12±0.9	12.2±1.1	14.2±1.2	14.0±1.2	14.3±1.1	14.5±1.3	14.3±1.0	14.4±1.0
		Stiffness (N/m)	269.1±26.6	259.7±29.3	267.1±30.3	185.4±38.8	183.8±39.6	187.5±38.4	251.4±27	248.6±25.5	253.4±24.8	223.1±48.3	220±40.4	218.6±37.5
	30°	Frequency (Hz)	14.5±1.0	14.1±0.9	14.4±1.0	12.0±0.9	12.0±0.7	12.1±1.0	13.9±0.9	13.7±0.9	14±1.0	17.0±1.9	16.9±1.9	16.7±1.8
		Stiffness (N/m)	259.3±24.5	254.3±25	260.6±29.7	186.6±35.9	191.6±30.1	195.4±34.8	250.1±25.8	247.2±24.1	251.8±22.0	388.9±96.6	386±99.3	372±92.6
	60°	Frequency (Hz)	15±1.2	14.6±0.9	15.1±1.3	13.7±1.3	13.8±0.9	13.7±1.1	15.2±0.9	15.3±0.8	15.3±1.1	21.0±3.4	21.4±2.7	21.3±2.7
		Stiffness (N/m)	272.7±28.9	268.1±25.4	274.8±29.8	243.1±39.4	248.6±33.4	245.8±35.1	293.5±27.1	295.5±25.1	296.9±29.1	603.8±146.8	631.6±120.6	619.2±122.6
	90°	Frequency (Hz)	15.2±1.4	14.9±1.1	15.4±1.3	15.2±1.4	15.4±1.4	15.5±1.6	16.3±1.2	16.4±1.1	16.3±1.2	24.3±3.5	24.5±3.2	24.6±3.4
		Stiffness (N/m)	276.8±33.5	275.6±29.9	283.6±32.8	289.8±45.2	296.3±45.6	296.4±48.3	330.2±34.8	330.2±29.5	330.1±33.7	713.5±127.3	720.3±125.9	714.7±132.4

SD = standard deviation, RF = rectus femoris, VM = vastus medialis, VL = vastus lateralis, PT = patellar tendon; Operator1 means recorded by GcC, Operator2 means recorded by JtW.
