# Supplementary material for: Reliability of a portable device for quantifying tone and stiffness of quadriceps femoris and patellar tendon at different knee flexion angles
Source: PLoS One. 2019 Jul 31;14(7):e0220521. doi: 10.1371/journal.pone.0220521 (PMC6668831; doi:10.1371/journal.pone.0220521)
Supplement: S3 Table — RF = Rectus Femoris, VM = Vastus Medialis, VL = Vastus Lateralis; PT = Patellar Tendon. #P<0.05 compare to 0°, *P<0.05 compare to 30°, Δ P<0.05 compare to 60°. (PDF) [file pone.0220521.s003.pdf]

Table 3. Mean values of three measurements and the difference at each angle

Location	Angles Of knee	Variable	RF	VM	VL	PT
			Mean value of Three measurements	Mean value of Three measurements	Mean value of Three measurements	Mean value of Three measurements
Dominant leg	0°	Frequency (Hz)	14.8	12.1	14.2	14.4
		Stiffness (N/m)	265.3	185.6	251.1	220.6
	30°	Frequency (Hz)	14.3	12.1	13.9	16.8#
		Stiffness (N/m)	258.1	191.2	249.7	382.3#
	60°	Frequency (Hz)	14.9	13.7*	15.3*	21.2#*
		Stiffness (N/m)	271.9	245.8*	295.3*	618.2#*
	90°	Frequency (Hz)	15.2	15.4*∆	16.3*∆	24.5#*∆
		Stiffness (N/m)	278.7	294.2*∆	330.2*∆	716.2#*∆
	P value	Frequency	0.186	0.00	0.00	0.00
		Stiffness	0.027	0.00	0.00	0.00
Non Dominant leg	0°	Frequency (Hz)	14.8	12.5	14.0	14.4
		Stiffness (N/m)	264.8	194.6	250.2	223.5
	30°	Frequency (Hz)	14.3	12.3	13.9	16.8#
		Stiffness (N/m)	259.4	199.9	250.2	385.4#
	60°	Frequency (Hz)	14.9	13.7*	15.3*	20.7#*
		Stiffness (N/m)	272.4	247.5*	294.2*	605.4#*
	90°	Frequency (Hz)	15.2	15.3*∆	16.4*∆	23.7#*∆
		Stiffness (N/m)	277.2*	293.5*∆	327.7*∆	690.1#*∆
	P value	Frequency	0.067	0.00	0.00	0.00
		Stiffness	0.045	0.00	0.00	0.00

RF = Rectus Femoris, VM = Vastus Medialis, VL = Vastus Lateralis; PT = Patellar Tendon. #$P<0.05$ compare to 0°, *$P<0.05$ compare to 30°, ∆ $P<0.05$ compare to 60°.
