# Supplementary material for: Reliability of a portable device for quantifying tone and stiffness of quadriceps femoris and patellar tendon at different knee flexion angles
Source: PLoS One. 2019 Jul 31;14(7):e0220521. doi: 10.1371/journal.pone.0220521 (PMC6668831; doi:10.1371/journal.pone.0220521)
Supplement: S4 Table — ICC = Intraclass Correlation Coefficients, CI = Confidence Intervals, RF = Rectus Femoris, VM = Vastus Medialis, VL = Vastus Lateralis; PT = Patellar Tendon (PDF) [file pone.0220521.s004.pdf]

Table 4. The results of Inter-operator ICC values and 95% CI

Location	Angles Of knee	Variable	RF ICC (95% CI)	VM ICC (95% CI)	VL ICC (95% CI)	PT ICC (95% CI)
Dominant leg	0°	Frequency (Hz)	0.90(0.48-0.96)	0.98(0.93-0.99)	0.98(0.95-0.99)	0.92(0.83-0.96)
		Stiffness (N/m)	0.92(0.75-0.97)	0.98(0.97-0.99)	0.97(0.94-0.99)	0.93(0.85-0.97)
	30°	Frequency (Hz)	0.89(0.66-0.95)	0.96(0.91-0.98)	0.92(0.84-0.96)	0.87(0.73-0.94)
		Stiffness (N/m)	0.94(0.87-0.97)	0.97(0.93-0.98)	0.92(0.82-0.96)	0.89(0.76-0.95)
	60°	Frequency (Hz)	0.96(0.92-0.98)	0.86(0.71-0.94)	0.85(0.69-0.93)	0.90(0.79-0.95)
		Stiffness (N/m)	0.98(0.95-0.99)	0.90(0.79-0.95)	0.89(0.76-0.95)	0.92(0.81-0.96)
	90°	Frequency (Hz)	0.95(0.90-0.98)	0.93(0.85-0.97)	0.89(0.78-0.95)	0.90(0.79-0.95)
		Stiffness (N/m)	0.97(0.94-0.99)	0.92(0.83-0.96)	0.84(0.64-0.93)	0.97(0.93-0.99)
Non Dominant leg	0°	Frequency (Hz)	0.90(0.69-0.96)	0.96(0.91-0.98)	0.99(0.97-0.99)	0.90(0.79-0.95)
		Stiffness (N/m)	0.91(0.70-0.97)	0.97(0.93-0.98)	0.97(0.94-0.99)	0.78(0.53-0.89)
	30°	Frequency (Hz)	0.90(0.40-0.97)	0.90(0.79-0.95)	0.81(0.61-0.91)	0.94(0.87-0.97)
		Stiffness (N/m)	0.94(0.86-0.97)	0.94(0.86-0.97)	0.94(0.88-0.97)	0.96(0.91-0.98)
	60°	Frequency (Hz)	0.87(0.54-0.95)	0.79(0.56-0.90)	0.90(0.78-0.95)	0.92(0.83-0.96)
		Stiffness (N/m)	0.96(0.90-0.98)	0.89(0.76-0.95)	0.88(0.75-0.94)	0.92(0.83-0.96)
	90°	Frequency (Hz)	0.94(0.83-0.98)	0.95(0.90-0.98)	0.97(0.93-0.98)	0.95(0.90-0.98)
		Stiffness (N/m)	0.98(0.96-0.99)	0.95(0.90-0.98)	0.95(0.90-0.98)	0.97(0.94-0.99)

ICC = Intraclass Correlation Coefficients, CI = Confidence Intervals, RF = Rectus Femoris, VM = Vastus Medialis, VL = Vastus Lateralis; PT = Patellar Tendon
