# Supplementary material for: Reliability of a portable device for quantifying tone and stiffness of quadriceps femoris and patellar tendon at different knee flexion angles
Source: PLoS One. 2019 Jul 31;14(7):e0220521. doi: 10.1371/journal.pone.0220521 (PMC6668831; doi:10.1371/journal.pone.0220521)
Supplement: S5 Table — ICC = Intraclass Correlation Coefficients, CI = Confidence Intervals, RF = Rectus Femoris, VM = Vastus Medialis, VL = Vastus Lateralis; PT = Patellar Tendon (PDF) [file pone.0220521.s005.pdf]

Table 5. The results of Intra-operator ICC values and 95% CI

Location	Angles Of knee	Variable	RF	VM	VL	PT
			ICC (95% CI)	ICC (95% CI)	ICC (95% CI)	ICC (95% CI)
Dominant leg	0°	Frequency (Hz)	0.72(0.48-0.86)	0.90(0.80-0.95)	0.87(0.75-0.94)	0.64(0.37-0.81)
		Stiffness (N/m)	0.81(0.63-0.90)	0.87(0.70-0.94)	0.90(0.81-0.95)	0.51(0.18-0.73)
	30°	Frequency (Hz)	0.75(0.54-0.87)	0.84(0.70-0.92)	0.72(0.49-0.86)	0.64(0.38-0.81)
		Stiffness (N/m)	0.76(0.56-0.88)	0.60(0.32-0.79)	0.49(0.16-0.72)	0.74(0.52-0.87)
	60°	Frequency (Hz)	0.84(0.69-0.92)	0.70(0.46-0.84)	0.83(0.67-0.92)	0.70(0.46-0.85)
		Stiffness (N/m)	0.87(0.73-0.93)	0.58(0.28-0.78)	0.69(0.44-0.84)	0.78(0.58-0.89)
	90°	Frequency (Hz)	0.83(0.67-0.92)	0.76(0.55-0.88)	0.84(0.65-0.93)	0.73(0.51-0.86)
		Stiffness (N/m)	0.82(0.65-0.91)	0.58(0.29-0.78)	0.64(0.34-0.81)	0.80(0.63-0.90)
Non Dominant leg	0°	Frequency (Hz)	0.78(0.58-0.89)	0.81(0.64-0.91)	0.58(0.30-0.78)	0.62(0.34-0.80)
		Stiffness (N/m)	0.70(0.46-0.85)	0.86(0.73-0.93)	0.79(0.65-0.90)	0.59(0.30-0.78)
	30°	Frequency (Hz)	0.74(0.53-0.87)	0.76(0.56-0.88)	0.70(0.45-0.84)	0.82(0.66-0.91)
		Stiffness (N/m)	0.70(0.45-0.84)	0.53(0.23-0.75)	0.41(0.06-0.67)	0.80(0.62-0.90)
	60°	Frequency (Hz)	0.74(0.51-0.87)	0.78(0.59-0.89)	0.70(0.47-0.85)	0.83(0.67-0.91)
		Stiffness (N/m)	0.77(0.58-0.89)	0.55(0.24-0.76)	0.42(0.07-0.68)	0.85(0.71-0.93)
	90°	Frequency (Hz)	0.80(0.77-0.95)	0.84(0.68-0.92)	0.82(0.65-0.91)	0.87(0.75-0.94)
		Stiffness (N/m)	0.85(0.69-0.93)	0.60(0.31-0.79)	0.69(0.43-0.84)	0.90(0.81-0.95)

ICC = Intraclass Correlation Coefficients, CI = Confidence Intervals, RF = Rectus Femoris, VM = Vastus Medialis, VL = Vastus Lateralis; PT = Patellar Tendon
