# Supplementary material for: Reliability of a portable device for quantifying tone and stiffness of quadriceps femoris and patellar tendon at different knee flexion angles
Source: PLoS One. 2019 Jul 31;14(7):e0220521. doi: 10.1371/journal.pone.0220521 (PMC6668831; doi:10.1371/journal.pone.0220521)
Supplement: S6 Table — SEM = Standard Error of Measurements, MDC = Minimal Detectable Change, LOA = Limits of Agreement, RF = Rectus Femoris, VM = Vastus Medialis, VL = Vastus Lateralis, PT = Patellar Tendon (PDF) [file pone.0220521.s006.pdf]

Table 6. The results of Inter-operator SEM, MDC and 95% LOA

Location	Angles Of knee	Variable	RF					VM					VL					PT				
			mean	SEM	MDC	95% LOA		mean	SEM	MDC	95% LOA		mean	SEM	MDC	95% LOA		mean	SEM	MDC	95% LOA	
						Lower	Upper				Lower	Upper				Lower	Upper				Lower	Upper
	0°	Frequency (Hz)	14.7	0.4	1.1	-0.6	1.6	12.4	0.2	0.6	-0.5	0.8	14.0	0.2	0.6	-0.6	0.7	14.4	0.3	0.8	-1.0	1.0
		Stiffness (N/m)	264.3	7.7	21.3	-16.1	32.6	190.8	6.5	18.0	-21.3	24.3	250.5	4.6	12.8	-19.7	14.0	222.4	8.7	24.1	-36.7	30.1
	30°	Frequency (Hz)	14.3	0.3	0.8	-0.7	1.4	12.2	0.2	0.6	-0.7	0.8	13.9	0.3	0.8	-1.0	1.0	16.8	0.6	1.7	-1.7	2.4
Dominant leg		Stiffness (N/m)	259.3	6.0	16.6	-19.2	26.2	197.1	6.0	16.6	-24.1	27.3	251.1	7.0	19.4	-26.5	19.5	386.9	29.5	81.8	-93.3	126.2
	60°	Frequency (Hz)	14.8	0.2	0.6	-0.7	0.9	13.8	0.4	1.1	-1.4	1.2	15.3	0.4	1.1	-1.3	1.6	20.7	0.7	1.9	-3.1	2.3
		Stiffness (N/m)	272.2	3.8	10.5	-15.4	16.3	248.4	9.9	27.4	-40.1	34.7	294.2	8.8	24.4	-49.8	44.7	606.4	30	83.2	-133.9	87.9
	90°	Frequency (Hz)	15.0	0.3	0.8	-0.9	1.0	15.5	0.4	1.1	-1.5	1.1	16.3	0.4	1.1	-1.6	1.2	23.7	0.9	2.5	-2.7	2.2
		Stiffness (N/m)	276.8	4.8	13.3	-20.3	14.5	295.9	11.0	30.5	-47.2	31.1	325.4	11.5	31.9	-48.4	30.0	690.4	18.8	52.1	-78.3	73.4
	0°	Frequency (Hz)	14.7	0.4	1.1	-0.2	1.6	12.1	0.2	0.6	-0.6	0.9	14.1	0.1	0.3	-0.3	0.8	14.4	0.4	1.1	-1.2	1.5
		Stiffness (N/m)	264.4	8.4	23.3	-16.3	35.1	184.6	6.7	18.6	-26.0	29.2	250.0	4.5	12.5	-13.7	19.4	221.5	20.7	57.4	-72.1	78.2
	30°	Frequency (Hz)	14.3	0.3	0.8	-0.4	1.3	12.0	0.3	0.8	-1.0	1.0	13.8	0.4	1.1	-1.3	1.6	16.9	0.5	1.4	-1.7	2.0
Non Dominant leg		Stiffness (N/m)	256.8	6.0	16.6	-16.4	26.3	189.1	8.1	22.5	-36.3	26.3	248.7	6.1	16.9	-19.6	25.4	387.4	19.4	53.8	-78.0	83.8
	60°	Frequency (Hz)	14.8	0.4	1.1	-0.7	1.6	13.8	0.5	1.4	-1.9	1.7	15.2	0.3	0.8	-1.1	1.0	21.2	0.9	2.5	-3.7	2.8
		Stiffness (N/m)	270.4	5.4	15	-16.0	25.3	245.8	12.0	33.3	-50.8	39.9	294.5	9.0	24.9	-35.7	31.7	617.7	37.9	105.1	-162.3	106.7
	90°	Frequency (Hz)	15.1	0.3	0.8	-0.7	1.3	15.3	0.3	0.8	-1.3	1.0	16.4	0.2	0.6	-0.9	0.7	24.4	0.7	1.9	-3.1	2.7
		Stiffness (N/m)	276.2	4.5	12.5	-15.5	18.0	293.1	10.1	28	-42.4	29.5	330.2	7.1	19.7	-26.9	26.9	716.9	21.8	60.4	-89.6	76.0

SEM = Standard Error of Measurements, MDC = Minimal Detectable Change, LOA = Limits of Agreement, RF = Rectus Femoris, VM = Vastus Medialis, VL = Vastus Lateralis, PT = Patellar Ten
