# Supplementary material for: Reliability of a portable device for quantifying tone and stiffness of quadriceps femoris and patellar tendon at different knee flexion angles
Source: PLoS One. 2019 Jul 31;14(7):e0220521. doi: 10.1371/journal.pone.0220521 (PMC6668831; doi:10.1371/journal.pone.0220521)
Supplement: S7 Table — SEM = Standard Error of Measurements, MDC = Minimal Detectable Change, LOA = Limits of Agreement, RF = Rectus Femoris, VM = Vastus Medialis, VL = Vastus Lateralis, PT = Patellar Tendon (PDF) [file pone.0220521.s007.pdf]

Table 7. The results of Intra-operator SEM, MDC and 95% LOA

Location	Angles Of knee	Variable	RF					VM					VL					PT				
			mean	SEM	MDC	95% LOA		mean	SEM	MDC	95% LOA		mean	SEM	MDC	95% LOA		mean	SEM	MDC	95% LOA	
						Lower	Upper				Lower	Upper				Lower	Upper				Lower	Upper
Dominant leg	0°	Frequency (Hz)	14.9	0.6	1.7	-1.8	1.9	12.6	0.4	1.1	-1.2	0.9	14.0	0.4	1.1	-1.0	1.0	14.4	0.5	1.4	-1.6	1.4
		Stiffness (N/m)	267.2	12.6	34.9	-33.1	38.4	196.8	16.1	44.6	-52.1	31.3	249.4	7.9	21.9	-22.4	21.4	223.2	23.1	64	-69.5	60.1
	30°	Frequency (Hz)	14.4	0.5	1.4	-1.2	1.6	12.3	0.4	1.1	-1.2	1.0	13.9	0.4	1.1	-1.3	1.4	16.8	1	2.8	-2.2	3.0
		Stiffness (N/m)	260.3	13.0	36.0	-35.1	38.0	201.8	22.8	63.2	-48.6	33.0	248.9	12.3	34.1	-24.2	25.8	388.8	46.2	128.1	-116.4	141.8
	60°	Frequency (Hz)	14.9	0.5	1.4	-1.3	1.2	13.7	0.5	1.4	-1.2	1.5	15.4	0.4	1.1	-1.3	1.3	20.6	1.2	3.3	-3.4	3.2
		Stiffness (N/m)	272.6	10.1	28	-30.0	29.2	246.3	19.4	53.8	-33.7	36.6	293.6	14.9	41.3	-38.4	35.7	599.1	49.2	136.4	-148.4	131.3
	90°	Frequency (Hz)	15.0	0.5	1.4	-1.5	1.3	15.2	0.6	1.7	-1.5	2.0	16.3	0.5	1.4	-1.6	1.0	23.7	1.2	3.3	-3.6	3.3
		Stiffness (N/m)	276.7	12.0	33.3	-36.8	31.2	290.3	24.6	68.2	-48.2	54.7	326.5	18.3	50.7	-48.9	26.0	689.4	47.3	131.1	-133.3	132.5
Non Dominant leg	0°	Frequency (Hz)	14.9	0.5	1.4	-1.4	1.6	12.2	0.5	1.4	-1.3	1.3	14.2	0.8	2.2	-2.3	2.0	14.4	0.7	1.9	-1.9	2.0
		Stiffness (N/m)	268.1	15.5	43	-41.6	45.7	186.5	14.3	39.6	-42.1	37.9	252.4	11.8	32.7	-34.9	31.1	220.9	27.5	76.2	-72.9	81.7
	30°	Frequency (Hz)	14.5	0.5	1.4	-1.2	1.6	12.1	0.5	1.4	-1.4	1.2	13.9	0.5	1.4	-1.6	1.4	16.8	0.8	2.2	-1.8	2.4
		Stiffness (N/m)	260.0	14.8	41	-43.3	40.6	191.0	24.2	67.1	-45.2	27.6	251.0	18.3	50.7	-38.3	34.9	380.4	42.1	116.7	-99.5	133.2
	60°	Frequency (Hz)	15.0	0.6	1.7	-1.8	1.7	13.7	0.6	1.7	-1.6	1.6	15.3	0.6	1.7	-1.7	1.4	21.1	1.3	3.6	-3.8	3.2
		Stiffness (N/m)	273.8	14	38.8	-41.1	37.0	244.5	24.8	68.7	-43.7	38.3	295.2	21.3	59.0	-50.3	43.5	611.5	52.0	144.1	-159.3	128.5
	90°	Frequency (Hz)	15.3	0.6	1.7	-1.9	1.4	15.4	0.6	1.7	-1.9	1.4	16.3	0.5	1.4	-1.5	1.5	24.5	1.2	3.3	-3.8	3.1
		Stiffness (N/m)	280.2	12.8	35.5	-41.1	27.6	293.1	29.4	81.5	-58.7	45.6	330.1	18.9	52.4	-42.2	42.4	714.1	40.7	112.8	-114.9	112.4

SEM = Standard Error of Measurements, MDC = Minimal Detectable Change, LOA = Limits of Agreement, RF = Rectus Femoris, VM = Vastus Medialis, VL = Vastus Lateralis, PT = Patellar Tendon
