# Supplementary material for: Reliability of a portable device for quantifying tone and stiffness of quadriceps femoris and patellar tendon at different knee flexion angles
Source: PLoS One. 2019 Jul 31;14(7):e0220521. doi: 10.1371/journal.pone.0220521 (PMC6668831; doi:10.1371/journal.pone.0220521)
Supplement: S1 Fig — (A) MyotonPRO device; (B) Identification of measurement angle; (C) Measurement with MyotonPRO. (PDF) [file pone.0220521.s008.pdf]

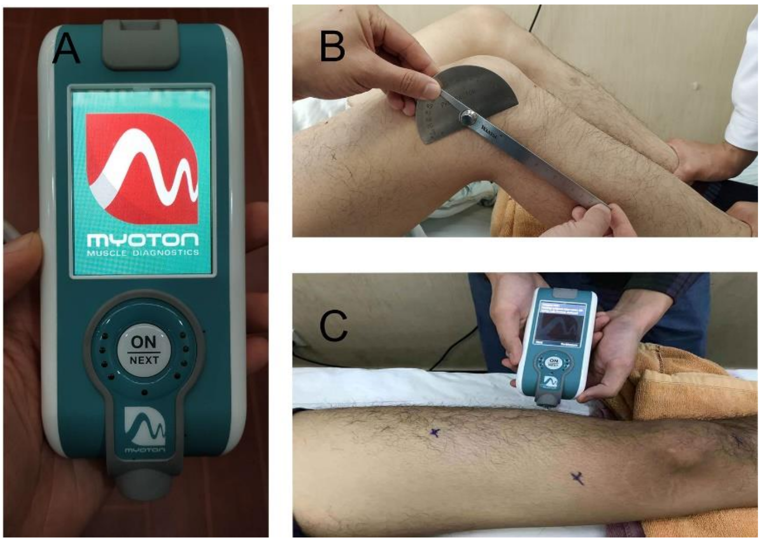


**S1 Fig. The MyotonPRO measurement technique. (A) MyotonPRO device; (B) Identification of measurement angle; (C) Measurement with MyotonPRO.**
