# Supplementary material for: Reliability of a portable device for quantifying tone and stiffness of quadriceps femoris and patellar tendon at different knee flexion angles
Source: PLoS One. 2019 Jul 31;14(7):e0220521. doi: 10.1371/journal.pone.0220521 (PMC6668831; doi:10.1371/journal.pone.0220521)
Supplement: S2 Fig — Examples Bland–Altman plots for MyotonPRO measurement of dominant rectus femoris tone(A) and stiffness(B) of healthy males. 95% limits of agreement and mean difference marked with dotted(––) and solid (—)lines. (PDF) [file pone.0220521.s009.pdf]

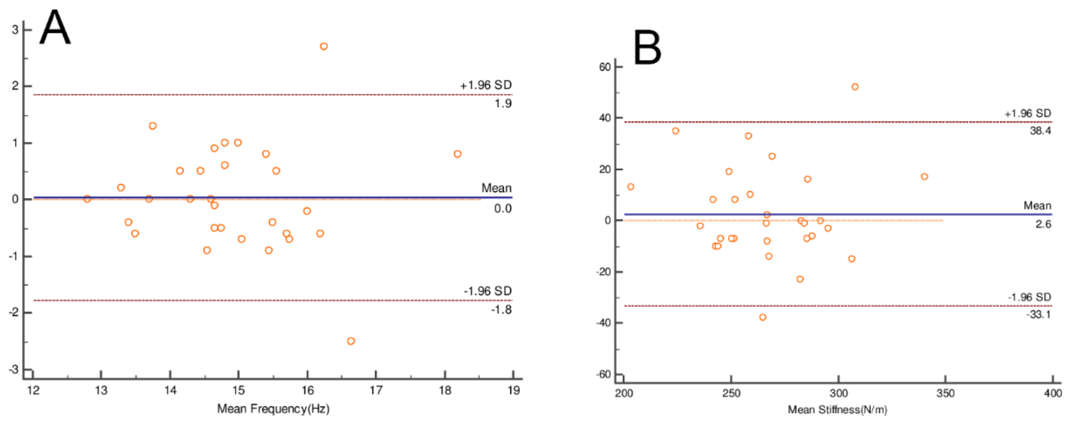

S2 Fig. Examples Bland–Altman plots for MyotonPRO measurement of dominant rectus femoris tone(A) and stiffness(B) of healthy males. 95% limits of agreement and mean difference marked with dotted(– –) and solid (—)lines.
